# Supplementary material for: Newborns’ brains Detect Foreign Rhythm in Native‐language Speech
Source: Dev Sci. 2026 Jul 17;29(5):e70254. doi: 10.1111/desc.70254 (PMC13377550; doi:10.1111/desc.70254)
Supplement: Supplementary file 1 — Supporting Information: desc70254‐supp‐0001‐SuppMat.pdf [file DESC-29-e70254-s001.pdf]

Supplementary information for article Detecting foreign rhythm in native-language speech at birth

1. Demographic and clinical background of included newborns

Participant ID	GA at birth	Weight at birth [g]	Sex	Ethnicity	Race	Education Mother	Education Father	estimated household income [per year, in USD]	Settlement type
1609	35+6	2160	F	Roma	white	Elementary	Elementary	20,500	Small town
1770	39+4	3260	F	Czech	white	Tertiary	Secondary	27,000	Village
1777	39+2	3180	F	Czech	white	Tertiary	Tertiary	34,500	Village
3018	41+3	3760	F	Czech	white	Tertiary	Secondary	32,900	County seat town
3034	39+5	4820	M	Czech	white	Secondary	Secondary	39,100	Village
3557	39+6	3830	M	Czech	white	Tertiary	Tertiary	22,900	County seat town
3707	40+1	3290	M	Czech	white	Tertiary	Secondary	30,000	Village
3818	38+2	2990	F	Czech	white	Tertiary	Secondary	48,300	County seat town
3980	39+5	4250	F	Czech	white	Tertiary	Tertiary	47,600	County seat town
4113	36+5	2340	M	Czech	white	Secondary	Secondary	27,200	Village
4313	39+6	3010	M	Czech	white	Elementary	N/A	9,300	County seat town
4642	39+6	3570	F	Czech	white	Tertiary	Tertiary	28,500	Village
4670	39+3	3640	M	Czech	white	Secondary	Secondary	26,700	Village
4684	40+2	3630	M	Czech	white	Secondary	Secondary	25,000	Village

5080	39+6	3330	F	Czech	white	Secondary	Secondary	23,500	Small town
5275	41+0	2520	F	Czech	white	Tertiary	Secondary	22,500	Small town
5569	39+5	3410	F	Czech	white	Secondary	Secondary	31,100	Village
5799	41+4	4070	F	Czech	white	Secondary	Secondary	34,500	Village
5904	37+2	3370	F	Czech	white	Secondary	Secondary	39,000	Small town
5951	39+2	3330	F	Czech	white	Tertiary	Tertiary	35,000	Village
5956	39+5	3570	M	Czech	white	Secondary	Secondary	23,500	Village
5971	39+0	3250	F	Czech	white	Tertiary	Secondary	42,800	County seat town
6193	39+1	2990	M	Czech	white	Secondary	Secondary	27,000	Village
6359	39+0	3430	F	Czech	white	Tertiary	Secondary	27,300	Village
6413	40+3	3490	M	Czech	white	Secondary	Secondary	65,600	Village
6478	38+4	3100	M	Czech	white	Tertiary	Secondary	31,100	County seat town
6572	39+3	3520	F	Czech	white	Secondary	Tertiary	32,500	Village
6615	39+4	3250	M	Czech	white	Tertiary	Secondary	36,000	County seat town
6656	38+4	3490	M	Czech	white	Secondary	Tertiary	28,500	Village
6727	39+2	4050	F	Czech	white	Tertiary	Tertiary	34,500	County seat town
6858	41+3	3370	F	Czech	white	Tertiary	Secondary	37,000	County seat town
7069	40+2	3940	M	Czech	white	Secondary	Tertiary	27,700	Village
7190	41+2	4280	M	Czech	white	Tertiary	Secondary	32,700	Small town
7300	40+1	3630	M	Czech	white	Secondary	Secondary	29,000	County seat town

7447	40+3	3350	M	Czech	white	Tertiary	Secondary	32,700	Village
7523	40+6	3320	M	Czech	white	Tertiary	Tertiary	30,000	Small town
7534	38+4	2930	F	Czech	white	Secondary	Secondary	24,200	Village
7805	39+6	3470	M	Czech	white	Secondary	Secondary	25,900	Village
8371	35+6	2430	M	Roma	white	Elementary	Elementary	20,500	Small town
8593	39+3	3610	F	Czech	white	Secondary	Secondary	41,000	Small town
8758	39+6	3590	F	Czech	white	Tertiary	Secondary	27,300	Village
9365	39+0	2810	F	Czech	white	Secondary	Secondary	39,000	Small town
9540	41+6	3750	M	Czech	white	Tertiary	Secondary	38,500	Village
9803	39+0	3400	F	Czech	white	Secondary	Tertiary	22,000	County seat town

Table 1: Clinical and demographic background of the newborn participants and their parents. Household income has been estimated based on parental profession and region of residence using the data of the Czech Statistical Office (Český statistický úřad, 2025).

2. HbR model summary

Effect	Estimate	SE	df	t	p
Intercept	-0.0048	0.0074	38.42	-0.65	.519
Condition (Native vs. Foreign)	0.0107	0.0064	31.48	1.66	.106
Hemisphere (Left vs. Right)	0.0006	0.0035	24.20	0.18	.860
Region (Front vs. Centro-temporal)	-0.0004	0.0030	32.52	-0.13	.901
Age (z-scored)	-0.0012	0.0073	46.11	-0.16	.872
Condition × Hemisphere	0.0018	0.0024	79.07	0.75	.457
Condition × Region	0.0037	0.0025	49.66	1.51	.138
Hemisphere × Region	-0.0015	0.0027	34.36	-0.55	.587
Condition × Hemisphere × Region	0.0005	0.0027	38.27	0.20	.844

Table 2: HbR model summary.

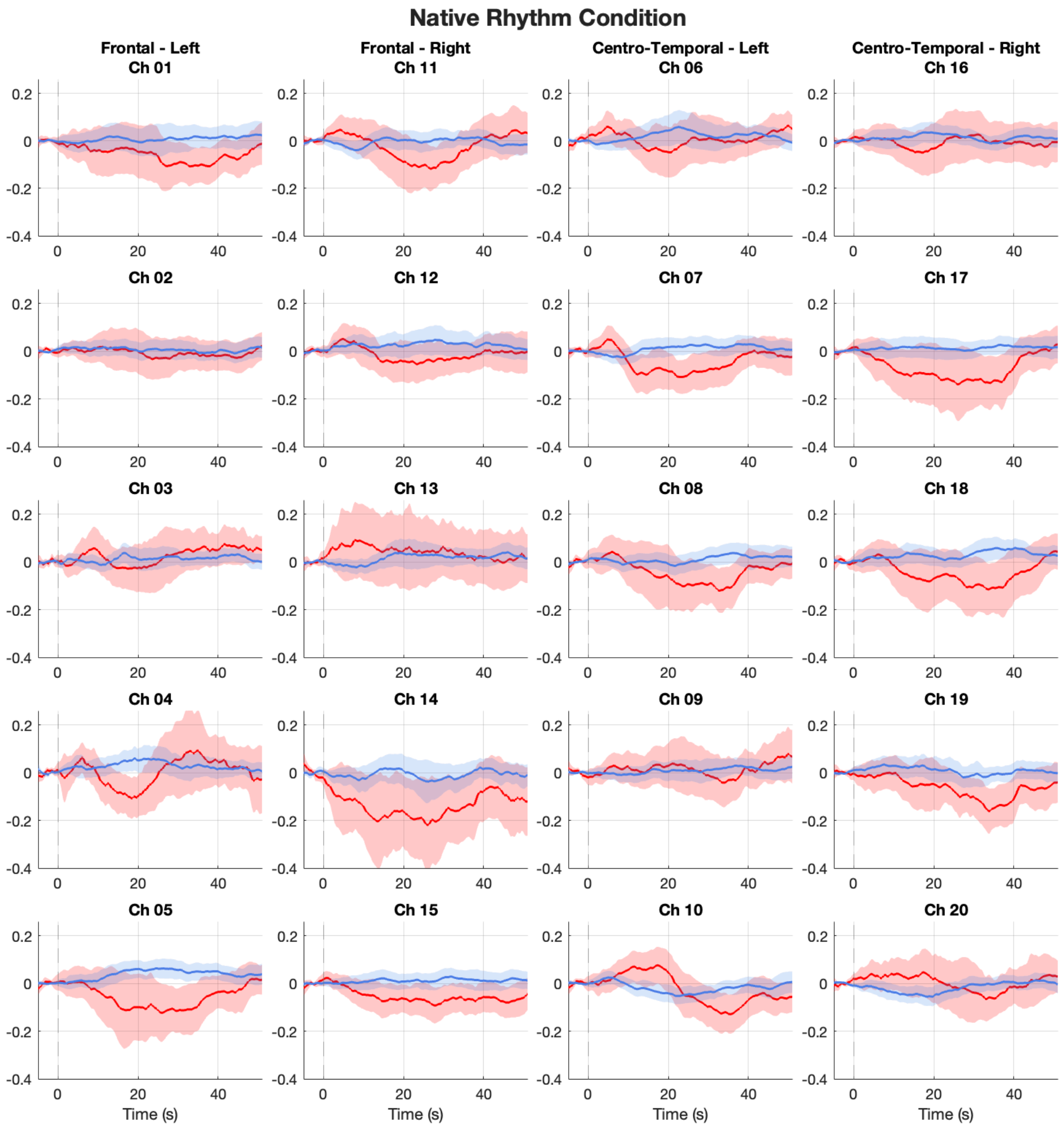

Figure 1. Grand-average HbO and HbR responses for the native-rhythm conditions, shown separately for each channel. The red line represents the HbO response, and the blue line represents the HbR response; shaded ribbons indicate 95% confidence intervals. A 51-second window (corresponding to the maximum stimulus duration plus the longest inter-trial silence) is displayed to better capture the temporal dynamics of the responses. This extended interval is included for visualization purposes only.

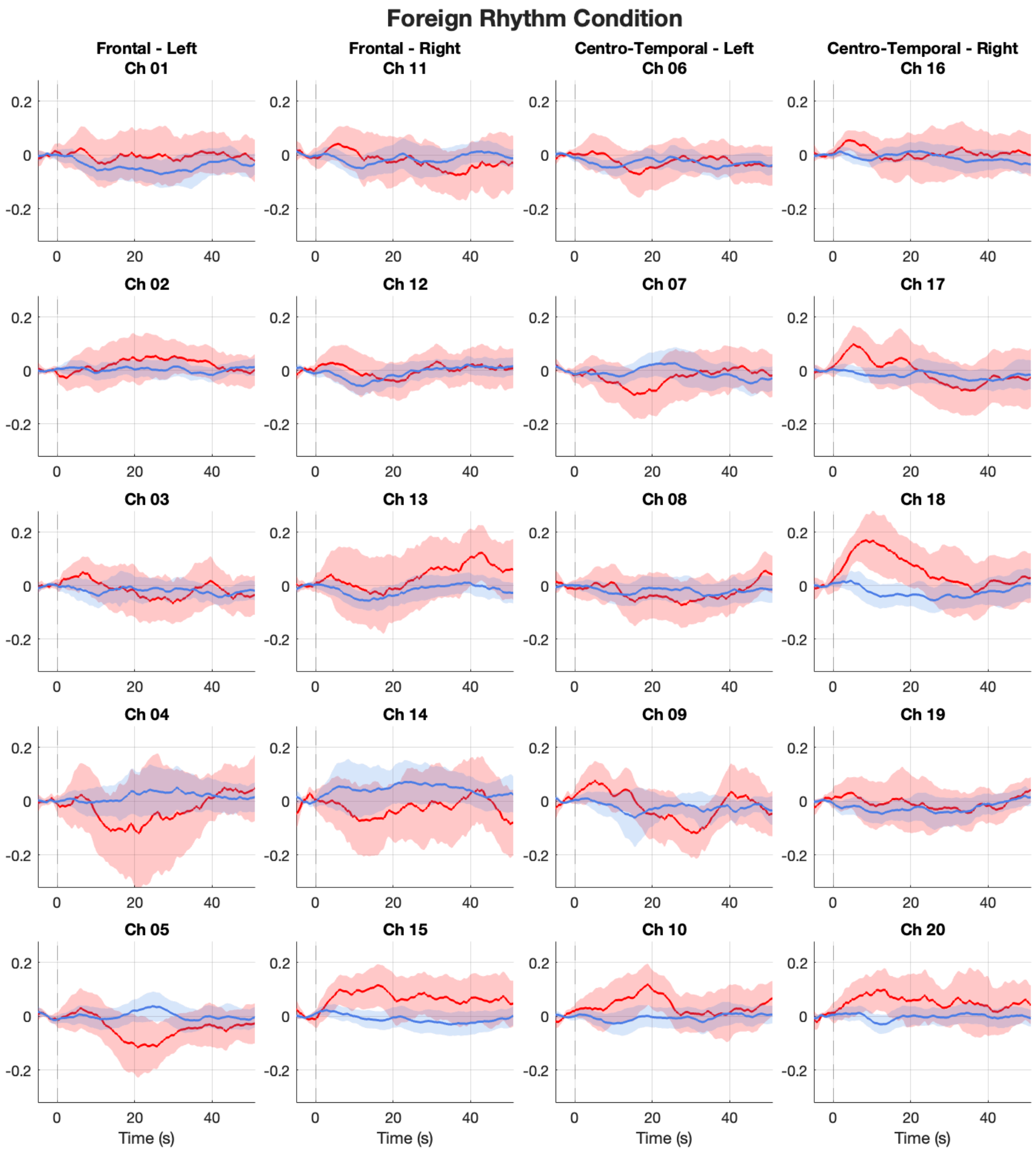

Figure 2. Grand-average HbO and HbR responses for the foreign-rhythm conditions, shown separately for each channel. The red line represents the HbO response, and the blue line represents the HbR response; shaded ribbons indicate 95% confidence intervals. A 51-second window (corresponding to the maximum stimulus duration plus the longest inter-trial silence) is displayed to better capture the temporal dynamics of the responses. This extended interval is included for visualization purposes only.
